# Drugs Repurposing Using QSAR, Docking and Molecular Dynamics for Possible Inhibitors of the SARS-CoV-2 M^pro^ Protease

**DOI:** 10.3390/molecules25215172

**Published:** 2020-11-06

**Authors:** Eduardo Tejera, Cristian R. Munteanu, Andrés López-Cortés, Alejandro Cabrera-Andrade, Yunierkis Pérez-Castillo

**Affiliations:** 1 Grupo de Bio-Quimioinformática, Universidad de Las Américas, Quito 170513, Ecuador; raul.cabrera@udla.edu.ec (A.C.-A.); yunierkis.perez@udla.edu.ec (Y.P.-C.); 2 Facultad de Ingeniería y Ciencias Aplicadas, Universidad de Las Américas, Quito 170513, Ecuador; 3 Faculty of Computer Science, Centre for Information and Communications Technology Research (CITIC), University of A Coruna, 15007 A Coruña, Spain; 4 Biomedical Research Institute of A Coruña (INIBIC), University Hospital Complex of A Coruna (CHUAC), 15006 A Coruña, Spain; 5 Centro de Investigación Genética y Genómica, Facultad de Ciencias de la Salud Eugenio Espejo, Universidad UTE, Quito 170129, Ecuador; aalc84@gmail.com; 6 Latin American Network for Implementation and Validation of Clinical Pharmacogenomics Guidelines (RELIVAF-CYTED), 28029 Madrid, Spain; 7 Carrera de Enfermería, Facultad de Ciencias de la Salud, Universidad de Las Américas, Quito 170513, Ecuador; 8 Escuela de Ciencias Físicas y Matemáticas, Universidad de Las Américas, Quito 170513, Ecuador

**Keywords:** SARS-CoV-2, COVID-19, QSAR, drugs repurposing, molecular dynamics

## Abstract

Wuhan, China was the epicenter of the first zoonotic transmission of the severe acute respiratory syndrome coronavirus clade 2 (SARS-CoV-2) in December 2019 and it is the causative agent of the novel human coronavirus disease 2019 (COVID-19). Almost from the beginning of the COVID-19 outbreak several attempts were made to predict possible drugs capable of inhibiting the virus replication. In the present work a drug repurposing study is performed to identify potential SARS-CoV-2 protease inhibitors. We created a Quantitative Structure–Activity Relationship (QSAR) model based on a machine learning strategy using hundreds of inhibitor molecules of the main protease (M^pro^) of the SARS-CoV coronavirus. The QSAR model was used for virtual screening of a large list of drugs from the DrugBank database. The best 20 candidates were then evaluated in-silico against the M^pro^ of SARS-CoV-2 by using docking and molecular dynamics analyses. Docking was done by using the Gold software, and the free energies of binding were predicted with the MM-PBSA method as implemented in AMBER. Our results indicate that levothyroxine, amobarbital and ABP-700 are the best potential inhibitors of the SARS-CoV-2 virus through their binding to the M^pro^ enzyme. Five other compounds showed also a negative but small free energy of binding: nikethamide, nifurtimox, rebimastat, apomine and rebastinib.

## 1. Introduction

Wuhan, China was the epicenter of the first zoonotic transmission of the severe acute respiratory syndrome coronavirus clade 2 (SARS-CoV-2) in December 2019 [1], and it is the causative agent of the novel human coronavirus disease 2019 (COVID-19) [2]. The outbreak of COVID-19 was declared as a Public Health Emergency of International Concern in January 2020, and as a pandemic in March 2020 by the World Health Organization (WHO) [3]. Nowadays, the geographical distribution of COVID-19 covers all continents except Antarctica, with millions of confirmed cases and over one million deaths in more than 190 countries worldwide [4].

The family *Coronavididae* is made up of seven human coronaviruses that are primarily respiratory pathogens: OC43, 229E, KHU1, NL63, Middle East Respiratory Syndrome Coronavirus (MERS-CoV), SARS-CoV, and SARS-CoV-2 [5]. The last three are members of the genus *Betacoronavirus*, which are characterized by causing mild to severe respiratory diseases, and having high mutation rates that result in viral genetic diversity, plasticity, and adaptability to invade a wide range of hosts [6].

The first SARS-CoV-2 genome (Wuhan-Hu-1; NC_045512) was sequenced in China in January 2020 [7,8]. This novel human coronavirus is a positive-sense single-stranded (ss) RNA virus of about 28–30 kilobases in length [8,9]. Its genomic structure is comprised of a 5′-untranslated region (UTR), 14 open reading frames (ORFs) encoding 29 proteins, and a 3′-UTR poly-A tail [10]. Additionally, the 16 non-structural proteins (nsps) encoded by the ORF1a and ORF1ab largest genes are involved in suppression of host immune responses, viral replication, the main protease (Mpro) or 3-chymotrypsin-like protease (3CL^pro^) [11], the ssRNA binding, the methyltransferase activity, the RNA-dependent RNA polymerase (RdRp) [12], the helicase activity, the exonuclease activity, the uridine-specific endoribonuclease and the RNA-cap methyltransferase [13]. On the other hand, genes of the 3′ terminus encode structural proteins such as the nucleocapside (N) protein, the membrane (M) glycoprotein, the envelope (E) protein, the spike (S) glycoprotein, and several accessory proteins [10,14].

The COVID-19 disease is caused when SARS-CoV-2 enters into human cells (i.e., nasal goblet secretory cells, lung type II pneumocytes or ileal absorptive enterocytes) [3] and exploits the host machinery for its own replication and spread [6]. The novel coronavirus entry is mediated by the S glycoprotein that forms homotrimers protruding from the viral surface [15]. The S glycoprotein has two subunits (S1 and S2). Six amino acids (L455, F486, Q493, S494, N501, and Y505) from the receptor-binding domain (RBD) of the S1 subunit directly bind to the peptide domain of angiotensin-converting enzyme 2 (ACE2) human receptor protein [16,17,18], which participates in the maturation of angiotensin controlling vasoconstriction and blood pressure [19]. In addition, the S glycoprotein is cleaved by the transmembrane serine protease (TMPRSS2) in a functional polybasic cleavage site at the S1-S2 boundary flanked by O-linked-glycans [15,20].

The 5’ terminal ORF1ab of SARS-CoV-2 encodes two viral replicase polyproteins, PP1a and PP1b. Both proteins are subsequently cleavage in a maturation process and produce 16 nsps, necessary for viral gene expression and replication [21]. This process is mediated by a main protease M^pro^, and by one or two papain-like proteases (PL^pro^) [22,23].

M^pro^ is a ~306 amino acid (aa) long protease that has similar cleavage-site specificity to that of picornavirus 3C protease, which is why it is also known as 3CL^pro^ [24]. It is interesting to note that M^pro^ is highly conserved among coronaviruses, both in peptide sequences and in 3D structures. Its 3D structure consists of three domains. The domains I and II are essentially beta-barrels, whereas domain III is mainly formed by alpha-helices. Domains I and II contain the conserved His41 and Cys145 catalytic dyad where Cys acts as nucleophile while His acts as a proton acceptor. In addition, there are two deeply buried subsites (S1 and S2) and three shallow subsites (S3–S5) in M^pro^. The S1 and S2 sites are involved in hydrophobic and electrostatic interactions with the enzyme’s substrates, while the shallow subsites S3–S5 can tolerate different functionalities [25,26,27].

Given the rapid spread of COVID-19 and its relatively high mortality, discovering coronavirus-specific drugs is urgent [28]. To date, there are several studies focusing on drug repurposing against SARS-CoV-2 proteins and COVID-19 symptoms [29,30,31]. Gordon et al. [13] identified druggable human proteins or host factors targeted by 69 compounds. López-Cortés et al. [3] identified immune system proteins targeted by 45 approved drugs. Zhou et al. [30] offered a powerful network-based methodology for the identification of potential drug combinations targeting SARS-CoV-2. Ahmad et al. [32] identified drugs binding strongly within the binding pockets of RdRp. Lastly, Kumar et al. [31] proposed that three inhibitors lopinavir-ritonavir, tipranavir, and raltegravir could bind to and inhibit M^pro^.

Since the M^pro^ protease is an essential protein within the viral cycle, its inhibitors would block viral replication. Earlier and recent repurposing studies have described possible candidates against both SARS-CoV and MERS-CoV [33,34]. Subsequently, two HIV-1 protease inhibitors had been identified as possible candidates for M^pro^ inhibition, namely lopinavir and ritonavir [35].

The goal of the presented work is to obtain drug candidates potentially inhibiting the M^pro^ protein of SARS-CoV-2. However, there are limited data available for inhibitors of this protein in SARS-CoV-2. On the other hand, several compounds are reported as confirmed inhibitors of M^pro^ in SARS-CoV. Considering this, and the high similarity between SARS-CoV and SARS-CoV-2 M^pro^ protease in terms of sequence and structure [34], our strategy was to initially construct a QSAR model for the screening of SARS-CoV M^pro^ inhibitors. The best candidates identified by the QSAR-based virtual screening were then evaluated using docking and molecular dynamics simulations using M^pro^ protease of SARS-CoV-2 as molecular target. The idea of exploring molecules with both activities to the M^pro^ of SARS-CoV as well as SARS-CoV-2 has also been explored by Gurung et al. [34]. These authors performed a comparison between M^pro^ in SARS-CoV, MERS-CoV and SARS-CoV-2 and carried out a virtual screening of possible drugs for each target using docking. Therefore, M^pro^ from SARS-CoV was not used as an initial screening and did not carry out molecular dynamic simulation. 

## 2. Results

### 2.1. QSAR Modelling Results

Traditionally, QSAR models are based on pre-calculated molecular features (feature engineering) and they are obtained with traditional Machine Learning methods. The deep learning revolution that has become a state-of-the-art in image and text classification is transforming chemoinformatics, too. Therefore, it is possible to not calculate any molecular feature but to convert the molecules into molecular graphs and to let the graph convolutional networks extract from the dataset the best molecular features for a specific task/classification. So, the current dataset was transformed into molecular graphs (with DeepChem function ConvMolFeaturizer) that were the inputs for the Graph convolutional networks (DeepChem function GraphConvModel). Using a split of train-test subsets of 80–20% results into 176 molecules for the training subset and 44 into the test subset (train_subset.txt and test_subset.txt in datasets folder, GitHub repository, respectively). The best model for inhibitors of M^pro^ of SARS-CoV provide an area under the receiver operating characteristics (AUROC) = 0.914 when evaluated in the test dataset. Additional statistics for the training and test subsets are presented in Table 1: accuracy, precision, AUROC and the area under the precision-recall curve (PRC-AUC).

**Figure 1:**
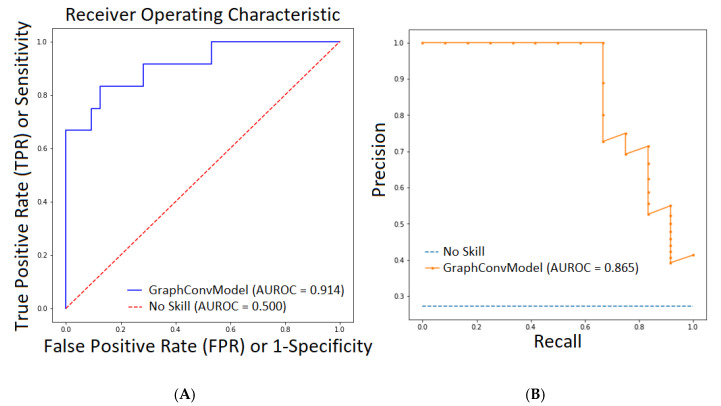
(**A**). AUROC plot defined by True Positive Rate (TPR) vs. False Positive Rate (FPR) (Sensitivity vs. Specificity) for our model (GraphConvModel) compared to a No Skill or random model. (**B**) PRC-AUC plot defined by Precision vs. Recall for our model (GraphConvModel) compared with a No Skill or random model.

Figure 1 (left) plots the ROC curve using Sensitivity vs. 1-Specificity for different probability cutoffs to represent the model performance. It can be observed that our model was far from a no skill (random) model (AUROC = 0.5). In order to be more precise, we plotted the second graph with the PRC-AUC curve (see Figure 1, Right) that depicts the precision vs. recall for different probability cutoffs compared with the no skill (random) model. A no skill or random model for AUC curve means that the model was not able to increase the True Positive Rate (TPR) compared with False Positive Rate (FPR), thus maintaining TPR equal with FPR (it was not able to learn anything good, it predicted random values). In the case of PRC-AUC, a no skill or random model was the one that maintained the precision very low (more false positives) while increasing the recall values (less false negatives). Both AUROC and PRC-AUC scores demonstrated good performance of the GraphConvModel model with the test subset.

### 2.2. QSAR Virtual Screening, Docking and Molecular Dynamics

A total of 10,246 molecules have been screened for potential M^pro^ inhibitors using the QSAR model previously obtained (prediction subset in our scripts, DB_SMILES4prediction.csv in datasets folder, GitHub repository). Here we show the top ranked 20 drugs (Table 2) (the full list of ranked drugs is presented in Table S1, Supplementary Materials).

**Figure 2:**
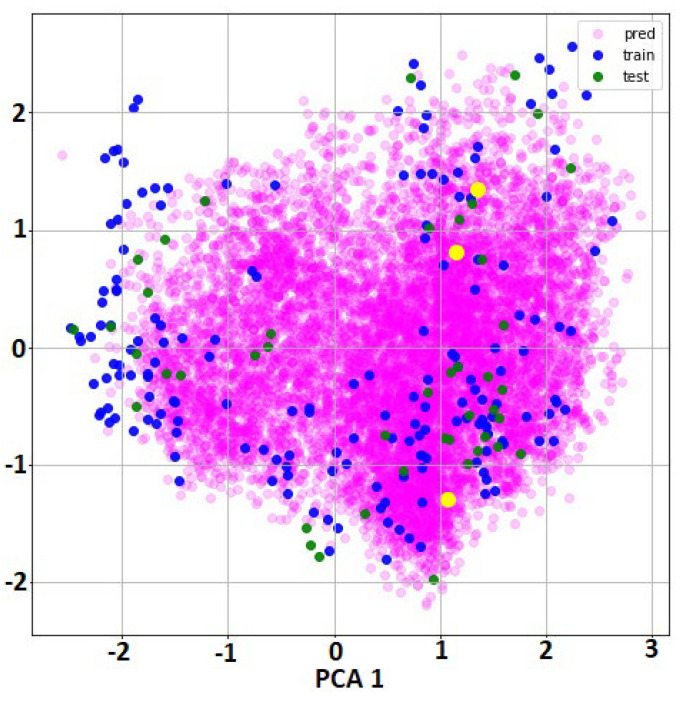
Two PCA components of the hidden features extracted by the GraphConvModel for training, test and prediction subsets. Green and blue marks correspond to the molecules used in training and test datasets for QSAR model construction. Purple marks correspond with all molecules used in virtual screening (DrugBank database) and yellow marks indicates the location of the best candidates obtained after molecular dynamic simulation: levothyroxine, amobarbital and ABP-700.

In order to check if the chemical space of the molecules in the prediction subset was close to the chemical space of the training and test subsets used in the QSAR model construction, we applied PCA analysis as represented in Figure 2. Because the inputs to this QSAR model were molecular graphs with 75 atomic properties for each atom in the molecule (it is variable across moleucles), it was not possible to use these local descriptors for this calculation. The current deep learning methodology is using the local initial molecular information to encode abstract internal representations that will be used in the next layers for the classification. Thus, we extracted from the GraphGather layer that hidden representation of all the molecules as a vector with dimension 32 * 256 (8192 features). In the second step, we reduced the feature dimensions to only two PCA components in order to present the molecules in the PCA space of the hidden features. It can be observed that the majority of our prediction molecules (those used in virtual screening from the DrugBank database) were inside the space of the training and test molecules. We included (yellow marks in Figure 2) the molecules that were selected as the best candidates of M^pro^ inhibitors after molecular dynamics simulation (levothyroxine, amobarbital and ABP-700) that will be discussed in the molecular dynamic simulation section.

It is important to note that Inositol nicotinate (Table 2) will produce nicotinic acid and inositol after hydrolysis; therefore, we include these molecules independently for docking and MD simulation. Moreover, Loitrix is formed by two molecules: levothyroxine (DB01583(2)) and liothyronine (DB01583(1)) and the two components were separately modeled.

**Figure 3:**
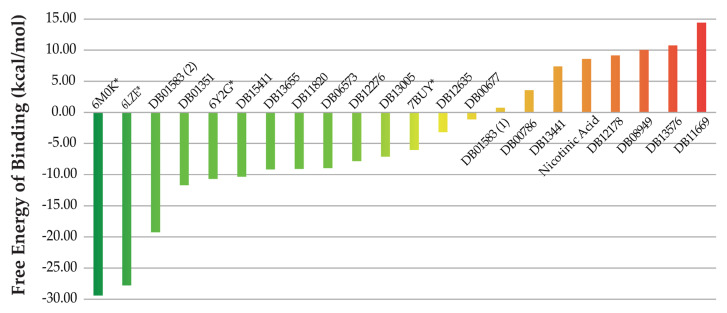
Estimated free energies of binding of the potential SARS-CoV-2 M^pro^ inhibitors. Levothyroxine (DB01583(2)), Amobarbital (DB01351), ABP-700 (DB15411), Nikethamide (DB13655), Nifurtimox (DB11820), Rebimastat (DB06573), Apomine (DB12276), Rebastinib (DB13005), Aleplasinin (DB12635), Isoflurophate (DB00677), liothyronine (DB01583(1)), Marimastat (DB00789), Niceritrol (DB13441), Telinavir (DB12178), Inositol nicotinate (DB08949), Aluminium nicotinate (DB13576) and Ortataxel (DB11669). The energies computed for the crystallographic complexes (PDB IDs 6LZE, 6M0K, 6Y2G and 7BUY) are marked with asterisks (*).

These top candidate molecules (see Table 2) were subject to molecular docking and molecular dynamics simulations. Among them, no possible binding mode was predicted for bismuth subcitrate potassium. The docking analysis of the remaining molecules leads to predicted complexes showing favorable ligand–receptor interactions and complementarity. The detailed results of the docking process are presented as Table S2 (Supplementary Materials). Among all predicted complexes, the best consensus docking score is obtained for Inositol nicotinate, followed by niceritrol, rebastinib, aleplasinin and liothyronine in the top five positions. On the other hand, the worst scored compounds are ortataxel, isoflurophate, nicotinic acid, aluminium nicotinate and amobarbital. Despite the fact that molecular docking is a widely used tool for virtual screening, the scoring functions used for these calculations neglect many important factors influencing molecular recognition and complexes’ stability in the sake of calculation speed. For this reason, the post-processing of the ligand–receptor complexes predicted with docking tools with more accurate methods based on the analysis of MD trajectories is recommended [36]. Such calculations allow for a better estimation of the free energy of binding of the ligands to the receptor. With this aim, MM-PBSA calculations were performed as described in the Material and Methods section. The predicted free energies of binding for the obtained docking complexes are summarized in Figure 3 and the detailed results are provided in Table S3 (Supplementary Materials).

For benchmarking purposes, four crystallographic structures of the SARS-CoV-2 M^pro^ enzyme in complex with known inhibitors were subject to the same protocol of MD simulations and MM-PBSA calculations as its potential inhibitors. In addition to the complex with an α-ketoamide inhibitor used for modeling studies (PDB ID 6Y2G), those reported with two peptide ligands (PDB IDs 6LZE and 6M0K) and with carmofur (PDB ID 7BUY) were studied [11,37,38]. These are marked with asterisks in Figure 3 and their detailed free energies of binding are available in Table S3 (Supplementary Materials). From the initial list of docked compounds, mecobalamin, hydroxocobalamin and drometrizole trisiloxane could not be used for MD simulations and hence discarded form any further analyses. This is due to the impossibility of parametrizing the cobalt ions present in Mecobalamin and Hydroxocobalamin, and the silicon atoms forming Drometrizole trisiloxane in Amber 18. The compound with the best free energy of binding is Levothyroxine, that is not among the five top docked compounds. On the other hand, the best compound, according to docking calculations (Inositol nicotinate), presents the third worst estimated free energy of binding. These observations highlight the importance of refining docking results with free energy simulations prior to selecting the best candidate M^pro^ inhibitors.

## 3. Discussion

The QSAR model obtained showed good prediction metrics. From the top 20 molecules selected for further analysis, all of them except bismuth subcitrate potassium (DB09275) were associated with favorable ligand–receptor interactions and complementarity (see Table S2, Supplementary Materials). Moreover, four out of the 20 molecules could not be studied with MD simulations because of parametrization issues. However, among the remained 16, eight drugs (50%): levothyroxine, amobarbital, ABP-700, nikethamide, nifurtimox, rebimastat, apomine and rebastinib showed a negative binding energy to M^pro^. The QSAR model was trained with compounds inhibiting another M^pro^ enzyme (even when similar to SAR-CoV-2 M^pro^ enzyme). Therefore, by using this type of model, additional validations (especially molecular dynamics) need to be done, in this case 50% of our predictions were corroborated by the computation of binding energies from MD simulations.

Liotrix is used as a replacement drug in hypothyroidism therapy and is composed by levothyroxine and liothyronine. Previous repurposing studies indicated that levothyroxine has an active effect against Trypanosoma cruzi proliferation (antichagasic activity) [39]. This is the compound with the lowest energy of binding and had been previously proposed by different in silico studies as candidate for COVID treatment [40,41], but using network based approaches. These previous studies do not focus in the drug-virus interaction but in the drug-host interactions which could potentially modulate the host response to viral infection. Moreover, liothyronine has a small positive binding energy to M^pro^, indicating that it as poor inhibitor candidate of the enzyme. This drug had been recently proposed as potential drug against SARS-CoV-2 by a different repurposing strategy against the RNA-dependent RNA polymerase enzyme [42]. Therefore, the potential interaction of both of these drugs with M^pro^ is a new report.

Nifurtimox, which is a drug with antichagastic activity, was also recently suggested through computational methods based on host protein network as possible drug against COVID in synergy with eflornithine [40]. Hence, levothyroxine and nifurtimox had been previously reported as potential candidates for COVID treatment and our calculations also indicate that these drugs are potential inhibitors of the SARS-CoV-2 replication through M^pro^ inhibition.

Amobarbital is a barbiturate related hypnotic and sedative drug unexplored in repurposing studies. Moreover, ABP-700 is a new drug under clinical trials, also as sedative (not chemically related to barbiturates). Similar to amobarbital, ABP-700 does not have any study related with any other pharmacological property. Etomidate is a very similar drug to ABP-700 and also used as sedative and hypnotic, had been previously probed to be antifungal [43] and recently repurposed as a possible influenza inhibitor considering its gene expression signature [44].

Interestingly several drugs in Table 2 are related to nicotinic acid: Inositol nicotinate, aluminium nicotinate, niceritrol and nikethamide. However, our MD analyses indicated that only nikethamide was predicted with negative free energy of binding. It is a stimulant of the respiratory system but we did not find any previous study related with its possible antiviral or even antibacterial activity.

Rebimastat is a broad matrix metalloproteinase inhibitor predicted to form a stable complex with M^pro^. Other virtual screening studies also indicate it as a possible inhibitor of the SARS-CoV-2 main protease [45]. Rebastinib is an inhibitor of the Tie2 tyrosine kinase and had been previously suggested as a potential drug for COVID-19 treatment using computational methods [46]. These consistencies with previous findings also increase the reliability of the potential use of both drug as inhibitors of SARS-CoV-2 replication through interaction with M^pro^.

On the other hand, the computation of the free energies of binding for marimastat, niceritrol, nicotinic acid, ortataxel, aluminium nicotinate and telinavir indicates that they are not good candidates for M^pro^ inhibition. Telinavir was identified by the QSAR model but the positive energy of binding lead to reject it as a potential inhibitor of the M^pro^ enzyme. This antiviral had been proposed as M^pro^ inhibitor by in-silico analyses from other authors [45,47]. However, the approaches employed in these two previous studies are different from ours. For example, no free energy of binding from MD simulations is computed in Eleftheriou et al. [47] while the MM-GBSA approach is employed by Durdagi et al. [45].

All the four known inhibitors of the M^pro^ enzyme from the SARS-CoV-2 virus used for benchmarking were predicted with negative values of free energy of binding. Among them, the two peptide inhibitors showed the lowest values of ΔG of binding. Interestingly, levothyroxine and amobarbital ranked after these two confirmed binders, before the α-ketoamide inhibitor co-crystallized with the receptor structure employed for modeling. Furthermore, eight of the potential M^pro^ inhibitors proposed in this investigation were predicted with better free energies of binding than the known carmofur inhibitor. Altogether, these results suggest that our top ranked potential M^pro^ inhibitors could form stable complexes with this enzyme.

**Figure 4:**
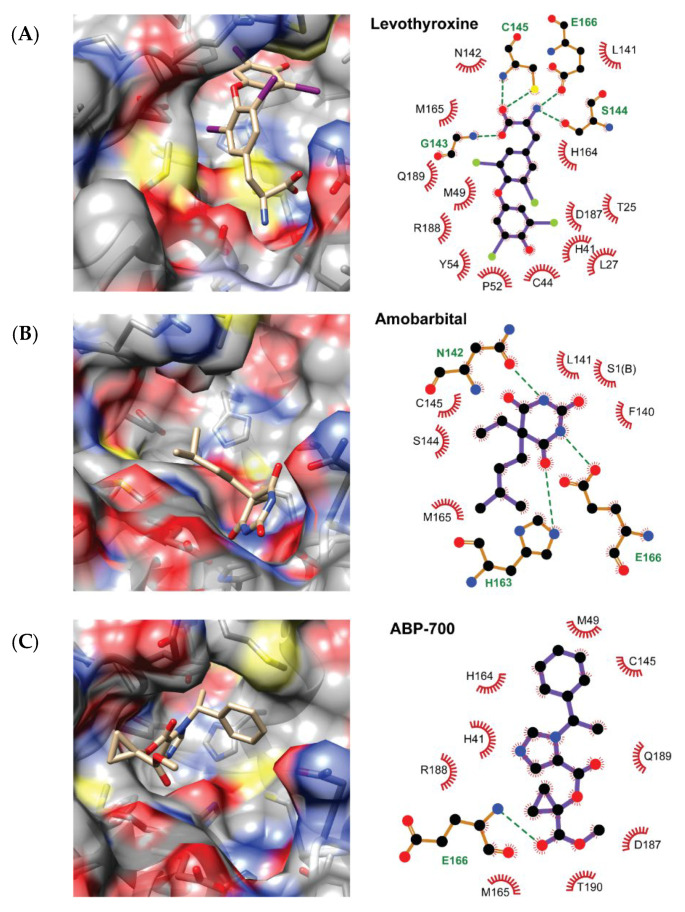
Predicted binding modes of Levothyroxine (**A**), Amobarbital (**B**) and ABP-700 (**C**) to the SARS-CoV-2 Mpro enzyme. The predicted hydrogen bonds between the ligands and the receptor are depicted using an all-atoms representation of the M^pro^ residues and dashed lines connecting them to the interacting ligand atoms. The color scheme is: black for carbon, red for oxygen, blue for nitrogen, yellow for sulfur and green for iodine.

Overall, the top three candidates for repurposing against M^pro^, according to our results, were levothyroxine, amobarbital and ABP-700. The predicted binding modes of these compounds to the M^pro^ enzyme are depicted in Figure 4. This figure also includes the diagram of the observed interactions between the compounds and the receptor. For depicting purposes, the 100 MD snapshots used for MM-PBSA calculations were clustered and the centroid of each cluster was selected as the representative ligand conformation. Only interactions observed in more than 50% of the studied MD snapshots are included in Figure 2. The figure was prepared with UCSF Chimera and LigPlot+ [48,49].

All the three compounds are predicted to bind inside the M^pro^ active site and to directly interact with the catalytic C145 amino acid, despite exploiting different binding regions within the cavity. Another common feature to them is the formation of hydrogen bonds with either the side chain or the backbone of E166. The interaction with C145 and the hydrogen bonding with E166 is also observed in the X-ray structures of the four SARS-CoV-2 inhibitors used here for benchmarking. Furthermore, these three candidate inhibitors partially overlap with the four known M^pro^ inhibitors included in our investigation. The latter mainly bind to the S1, S2 and S4 sub-pockets of the receptor [11,37,38], while none of these completely exploits the S1’ sub-pocket. In contrast, levothyroxine and ABP-700 are predicted to fully occupy the S1’ region. On the other hand, only ABP-700 interacts with the residues at the S4 region among our top three candidate inhibitors.

Levothyroxine is predicted to hydrogen bond the backbone and the side chain of C145, the backbone of G143 and the side chain of S144. Among these, the hydrogen bond to C145 is a common feature with the four benchmarking inhibitors, while the one with G143 is observed for all of them except in the 6LZE structure. The bi-p-iodine-phenyl ring of levothyroxine stacks in front of the second catalytic residue H41 and occupies the S1’ sub pocket lined by T25, C44, M49, P52, Y54 and D187. On the other hand, the central bi-iodine benzyl ring interacts with L27, H41, M165, R188 and Q189, with one of its iodine atoms projecting towards S2. The aminopropane moiety of levothyroxine is more exposed to the solvent than the rest of the molecule and mainly interacts with L141, N142, G143, S144, C145, H164 and E166 at the S1 region. The high shape complementarity between this compound and the active site of M^pro^, as well as the presence of hydrogen bonds with the receptor, and the predicted π-π stacking interaction with H41 might justify that it is the one with the lowest predicted ΔG of binding among all the evaluated chemicals.

Both amobarbital and ABP-700 are predicted to bind M^pro^ in conformations more exposed to the solvent than levothyroxine. Amobarbital is the only compound among our top three M^pro^ inhibitor candidates that does not interact with the catalytic H41 residue, even though it is predicted to block the access to C145. It is predicted to hydrogen bond N142, H163 and E166. The hydrogen bond to H163 is a shared interaction with all benchmarking inhibitors except carmofur. Additional interactions are observed with F140, L141, S144, M165 and S1 of the second enzyme monomer. The 1,3--diazinane--2,4,6--trione ring of amobarbital overlaps with the aminopropane moiety of levothyroxine. Finally, the benzyl ring of ABP-700 is predicted to overlap with the central ring of levothyroxine while preventing the access to the catalytic H41 and C145 residues of M^pro^. This molecule is expected to hydrogen bond the backbone of E166 and its imidazole ring points perpendicularly to the side chain of H41 at the S1’ region in a position favorable for the π-π stacking of these aromatic rings. The rest of the interactions of ABP-700 with the receptor occur with M49, H164, M165, D187, R188, Q189 and T190, spanning the S1’, S2 and S4 sub-cavities.

## 4. Materials and Methods

### 4.1. QSAR Modelling

All compounds with reported interactions to the CHEMBL5118 and CHEMBL3927 targets were extracted from the ChEMBL database. These references correspond to the replicase polyprotein 1ab and the SARS coronavirus 3C-like (M^pro^) proteinase, respectively. These were the only coronavirus-related targets in the ChEMBL database. A closer analysis to the targets and articles associated with the reported interactions showed that in both targets there were several compounds that interact with the M^pro^ protease domain which was the same for both targets. Therefore, all compounds with reported IC50 values for interactions with the M^pro^ protease in any of these targets were combined as a single modelling problem.

A second processing step of the data was performed as follows: (1) all interactions were classified as active if IC50 was lower than 10uM and inactive otherwise; (2) interactions with ambiguous reports, i.e., different assays reporting contradictory classifications, were removed. The final dataset comprises 229 interactions (70 active and 159 inactive).

DeepChem (python package) with Graph Convolutional Networks [50] for molecules was used to build a classifier for M^pro^ inhibitors and no inhibitors. Thus, the SMILES formula of molecules was transformed into molecular graphs that were populated to the Graph Convolutional Networks. In this way, there was no need for feature engineering, and the “kernels” of the convolutions were automatically learned from the dataset during the training. Featurization took place by using the Duvenaud graph convolutions [50]. The metrics for the training was the Area Under the Receiver Operating Characteristic curve (AUROC), the data were split into 80% training and 20% test subsets by using stratification.

The network-type classifiers predicts probabilities of a class, not the direct class. Thus, we can use any cutoff in order to decide the classes. Thus, the predicted probabilities could be interpreted by using different thresholds in order to allow the operator of the model to trade-off concerns in the model errors (ex: number of false positives vs. number of false negatives). In the case of classification, the performance of the model using a test subset is evaluated using accuracy, precision, recall or more complex metrics such as area under curve (AUC) of the receiver operating characteristic (ROC) curve (AUROC) [51] and the precision recall curve (PRC) or PRC-AUC [52]. The ROC curve shows True Positive Rate (TPR) (referred also as Sensitivity) versus the False Positive Rate (FPR) (referred also as 1-Specificity) for binary classifier when different probability thresholds are used. AUROC represents the area under the ROC curve, with values between 0 and 1 (1 is the best score, the model is more stable; 0.5 demonstrates a random model) and PRC-AUC correspond with the area under the precision-recall curve.

The PRC curve shows the tradeoff between precision and recall for the same binary classifier when the probability threshold is varied. When the classes are imbalanced, this is a good measure of success of prediction. High scores mean that the model is predicting accurate results (high precision), as well as returning a majority of all positive results (high recall). Precision was computed as TP/(TP + FP), where TP = number of true positives, FP = number of false positives. Recall is defined as TP/(TP + TN), where TN = number of true negatives. Accuracy is calculated as (TP + TN)/(TP + TN + FP + FN), where FN = number of false negatives. DeepChem has implemented AUROC as the default metric to test the quality of the classifier.

Different splits, seeds, dropouts, batch_sizes and training epochs were tested. The script (jupyter notebook with python), datasets, molecular features, the best classifier and the prediction results can be accessed as a free GitHub repository at https://github.com/muntisa/Anticoronavirals-Classifier-using-DeepChem. The script was executed into Google Colab using GPU support.

The main Jupyter notebook of the repository (antivirals_DeepChem.ipynb) presents all the details about the dataset, feature calculation, dataset split, model training and prediction for extra molecules. The information on the repository was organized as follows:

- The entire dataset was available as antivirals_SMILES.csv in datasets folder of the repository (229 molecules as SMILES representation, antivirals_SMILES.csv in datasets folder).
- The external dataset used to predict anti-M^pro^ activity from drug repurposing was available as DB_SMILES4prediction.csv in the datasets folder of the repository (10,246 molecules with DB ID and SMILES formula).
- For all molecules from the full dataset and external set for predictions, specific features were calculated using DeepChem function ConvMolFeaturizer, an implementation of the Duvenaud graph convolutions that computed a vector of 75 local descriptors for each atom in a molecule. Thus, each molecule was represented as an array with dimension number of atoms*75. As consequence, the initial input features were graph representations, not vector of values (as in classical QSAR). There was no possibility to cluster the molecules using this type of information.
- We used 75 internal features for the convolutional graphs, batch size = 32 during 70 epochs and dropout = 0.05 as parameters for training with DeepChem function GraphConvModel. The optimization algorithm to find the best model was minimizing the error between the observed and predicted classes.
- The dataset was randomly split (seed = 80) into 80–20% train-test subsets using DeepChem function SingletaskStratifiedSplitter that divides the dataset keeping the same ratio of classes across the training and test subsets. The result were 176 molecules in the training subset (train_subset.txt in datasets folder) and 44 in the test subset (test_subset.txt in datasets folder).
- The training subset was used to build the best classifier using the two classes and the test subset was used to evaluate the model performance using AUROC as the performance metric. The training used a deterministic optimization and therefore it is possible to reproduce the same classifier. In addition, all the calculated features and the final model are available as files in a specific folder at the public repository (using specific DeepChem format).

We used the sklearn and pyplot packages from python to create the AUROC and PRC-ROC plots.

### 4.2. Virtual Screening

The QSAR model constructed for the inhibitors of the M^pro^ protease of SARS-CoV was used as the first stage in virtual screening of DrugBank molecules [53]. A total of 10,254 drugs from DrugBank were used for screening. Only the top 20 candidates were evaluated in docking and molecular dynamic simulation. In order to check if the predicted molecules are in the same chemical space used for QSAR model construction (the training and test molecules datasets), we used principal component analysis (PCA) representation with two components.

### 4.3. Molecular Docking

The X-ray structure of the SARS-CoV-2 main protease (M^pro^) in complex with an α-ketoamide inhibitor (PDB code 6Y2G) was used for molecular docking calculations [11]. One initial 3D conformer of each compound was generated with OpenEye’s Omega [54] (available: http://www.eyesopen.com) and partial atomic charges were added to them with Molcharge from OpenEye.

Docking was performed as described in previous publications [55] by using the Gold software [56]. The binding site was defined from the inhibitor co-crystalized with M^pro^ and the residues forming it were considered as flexible during calculations. The compounds were docked into one of the active sites of the M^pro^ dimer using the CHEMPLP scoring function. A total of 30 different docking solutions were explored for each ligand and they were further rescored with the ASP, ChemScore and GoldScore scoring functions of Gold. The search efficiency parameter of Gold was set to 200% for molecular docking.

The selection of the most probable binding modes of each compound to M^pro^ was carried out following the previously described consensus scoring methodology [55,57,58]. Consensus scoring took place independently for each compound to rank its 30 predicted binding modes. For each compound binding pose *i*, its consensus Z-score *i* (*Z_i_*) is computed as $Z_{i}=\frac{\sum_{j}\frac{S_{i,j}-\bar{S_{j}}}{std\left(S_{j}\right)}}{4}$, where *S_i_*_,*j*_ is the score of compound *i*, according to scoring function *j*, $\bar{S_{j}}$ the mean of scoring function *j* across all compound conformers and *std*(*S_j_*) the standard deviation of scoring function *j*. The compound conformer with the highest consensus score and any other one with *Z_i_* > 1 were selected for additional calculations.

### 4.4. Molecular Dynamics Simulations and Estimation of the Free Energies of Binding

All the possible ligand–receptor complexes predicted by the molecular docking approach were subject to molecular dynamics (MD) simulations and predictions of their free energies of binding. The complexes were prepared to contain one ligand at each active site of the SARS-CoV-2 Mpro. This was achieved by means of a home-made python script in two steps: (1) superimposition of the binding pocket containing the docked ligand into the one not explored during docking calculations, and (2) the extrapolation of the ligand and side chains conformers coordinates to the ligand free active site. MD simulations of these dimeric complexes proceeded with Amber 18 [59] as previously described [55,58].

Systems for MD simulations were parametrized with the *tleap* tool of Amber 18. They were embedded in truncated octahedron boxes, solvated with TIP3P water molecules, and excess charges were neutralized by the addition of either Na^+^ or Cl^−^ counterions. The Particle Mesh Ewald (PME) method was used to treat long-range electrostatic interactions. The MD ready systems were energy minimized in two steps, the first of which consisted in 500 steps of the steepest descent method followed by 500 cycles of conjugate gradient at constant volume. All atoms but solvent and counterions were restrained with a force constant of 500 kcal/mol·Å^2^ and the PME distance cutoff was set to 12 Å. The second energy minimization stage proceeded also at constant volume and included 1500 iterations of the steepest descent method and 1000 cycles of conjugate gradient. In addition, no restrain was applied in this step and the PME cutoff was set to 10 Å.

The energy minimized systems were gradually heated from 0 K to 300 K during 20 ps with a time step of 2 fs. All atoms except solvent and ions were constrained with a force constant of 10 kcal/mol·Å^2^ during heating. From this step on, all bonds involving hydrogen atoms were constrained with the SHAKE algorithm and the PME distance cutoff was set to 10 Å. In addition, during all MD steps temperature was controlled with a Langevin thermostat, setting the collision frequency of 1 ps^−1^. Afterward, the heated systems were equilibrated for 100 ps with a time step of 2 fs and constant temperature (300 K) and pressure (1 bar). Pressure was controlled with isotropic position scaling, setting the relaxation time to 2 ps. For each system, the final snapshot of the equilibration process was used as input to 10 MD simulations lasting 2 ns each, accounting for 20 ns of simulation time for each ligand–M^pro^ complex. These simulations were set up with different initial random velocities for a better exploration of the conformational space of the predicted complexes.

The free energies of binding of all compounds to the SARS-CoV-2 M^pro^ enzyme were predicted with the MM-PBSA method as implemented in Amber 18 [60]. A total of 100 snapshots, one every 200 ps, were extracted from the previously obtained MD trajectories for the estimation of the free energies of binding. The ionic strength for MM-PBSA calculations was set to 0.100 mM.

## 5. Conclusions

Our results indicate that from the 20 drug candidates previously screened using the QSAR model levothyroxine, amobarbital and ABP-700 are the best potential inhibitors of the SARS-CoV-2 M^pro^ enzyme. Other five compounds also showed negative but higher free energies of binding: nikethamide, nifurtimox, rebimastat, apomine and rebastinib. From this group, levothyroxine and nifurtimox had been previously proposed for COVID treatment based on protein interaction network analyses in the host and consequently as modulator of the response to viral infection. We are indicating here that those drugs can also directly interact with the M^pro^ enzyme of the SARS-CoV-2 virus. Moreover, rebimastat and rebastinib had also been previously suggested as potential inhibitors of the SARS-CoV-2 replication through their interactions with the M^pro^ enzyme. These consistencies with previous findings, as well as predicted free energies of binding comparable to those of confirmed inhibitors, increase the reliability of the potential use of our drug candidates for COVID treatment.

## Figures and Tables

**Table 1:** Statistics for the QSAR model obtained to predict inhibitors of M^pro^ of SARS-CoV.

Metrics	Train	Test
Accuracy	0.977	0.841
Precision	0.970	0.830
AUROC	0.998	0.914
PRC-AUC	0.995	0.865

Note: AUROC—area under the receiver operating characteristics; PRC-AUC—area under precision-recall curve.

**Table 2:** Top 20 drugs candidates from DrugBank as inhibitors of M^pro^ of SARS-CoV.

Name	Probability	DrugBank ID	Name	Probability	DrugBank ID
Inositol nicotinate	0.999	DB08949	Aluminium nicotinate	0.992	DB13576
Telinavir	0.998	DB12178	Amobarbital	0.991	DB01351
Ortataxel	0.998	DB11669	ABP-700	0.991	DB15411
Niceritrol	0.997	DB13441	Rebastinib	0.988	DB13005
Rebimastat	0.996	DB06573	Bismuth subcitrate potassium	0.987	DB09275
Apomine	0.994	DB12276	Drometrizole trisiloxane	0.987	DB11585
Mecobalamin	0.994	DB03614	Aleplasinin	0.985	DB12635
Nikethamide	0.993	DB13655	Liotrix	0.984	DB01583
Hydroxocobalamin	0.993	DB00200	Nifurtimox	0.983	DB11820
Marimastat	0.992	DB00786	Isoflurophate	0.982	DB00677

## Supplementary Materials

Table S1: Ranked drugs obtained from screening using QSAR model, Table S2: Results of the docking of the 20 top ranked compounds predicted by the QSAR model, Table S3: Estimated free energies of binding to the M^pro^ of SARS-CoV-2.
