# Supplementary material for: Drugs Repurposing Using QSAR, Docking and Molecular Dynamics for Possible Inhibitors of the SARS-CoV-2 Mpro Protease
Source: Molecules. 2020 Nov 6;25(21):5172. doi: 10.3390/molecules25215172 (PMC7664330; doi:10.3390/molecules25215172)
Supplement: Supplementary file 1 [file molecules-25-05172-s001.zip › Table S2.docx]

**Table S2. Results of the docking of the 20 top ranked compounds predicted by the QSAR model**

| Compuesto | Conformer | CHEMPLP | | GoldScore | | ChemScore | | ASP | | Consensus Z-Score |
| --- | --- | --- | --- | --- | --- | --- | --- | --- | --- | --- |
|  |  | Score | Z-Score | Score | Z-Score | Score | Z-Score | Score | Z-Score |  |
| DB08949 | 1 | 93.50 | 2.41 | 68.60 | 0.92 | 13.60 | 1.33 | 52.42 | 1.85 | 1.63 |
|  | 2 | 85.81 | 1.73 | 40.42 | 0.26 | 11.75 | 1.07 | 45.84 | 0.99 | 1.01 |
| DB12178 | 1 | 89.08 | 1.91 | 21.80 | 0.82 | 12.61 | 0.61 | 41.04 | 1.56 | 1.23 |
|  | 2 | 81.77 | 1.07 | 27.01 | 0.99 | 13.23 | 0.78 | 41.50 | 1.64 | 1.12 |
| DB11669 | 1 | 70.23 | 1.75 | -12.65 | 0.25 | -3.67 | 0.21 | 25.71 | 1.79 | 1.00 |
| DB13441 | 1 | 93.47 | 2.22 | 57.61 | 1.00 | 16.62 | 1.78 | 49.17 | 1.94 | 1.74 |
| DB06573 | 1 | 61.13 | 0.11 | 22.67 | -0.07 | 16.76 | 2.10 | 27.14 | 1.66 | 0.95 |
| DB12276 | 1 | 71.07 | 1.44 | 23.08 | 0.70 | 13.64 | 1.64 | 12.93 | 0.23 | 1.00 |
| DB13655 | 1 | 49.46 | 1.89 | 28.74 | 1.89 | 14.21 | 1.84 | 21.11 | 1.40 | 1.76 |
|  | 2 | 47.07 | 1.04 | 26.43 | 1.16 | 14.76 | 2.24 | 22.15 | 1.86 | 1.57 |
| DB00786 | 1 | 51.48 | 0.42 | 27.16 | 0.79 | 11.43 | 1.04 | 26.10 | 1.48 | 0.93 |
| DB13576 | 1 | 33.79 | 1.79 | 25.90 | 0.96 | 13.48 | 0.97 | 20.10 | 1.82 | 1.39 |
|  | 2 | 32.06 | 1.06 | 24.87 | 0.63 | 12.56 | 0.20 | 20.78 | 2.19 | 1.02 |
|  | 3 | 32.65 | 1.31 | 28.29 | 1.75 | 12.28 | -0.03 | 18.64 | 1.01 | 1.01 |
| DB01351 | 1 | 42.72 | 1.63 | 22.39 | 0.49 | 13.12 | 1.13 | 24.17 | 2.90 | 1.54 |
|  | 2 | 41.83 | 1.35 | 18.28 | -0.07 | 14.36 | 1.70 | 20.45 | 1.40 | 1.10 |
|  | 3 | 42.34 | 1.51 | 29.16 | 1.42 | 10.52 | -0.06 | 20.07 | 1.25 | 1.03 |
| DB15411 | 1 | 66.96 | 2.13 | 43.55 | 1.17 | 12.98 | 1.62 | 28.14 | 1.21 | 1.53 |
|  | 2 | 64.23 | 1.67 | 42.46 | 0.95 | 13.21 | 1.74 | 29.98 | 1.74 | 1.53 |
|  | 3 | 62.26 | 1.35 | 45.07 | 1.48 | 10.10 | 0.11 | 31.32 | 2.13 | 1.27 |
| DB13005 | 1 | 87.76 | 1.05 | 45.61 | 0.56 | 26.39 | 1.72 | 42.81 | 0.82 | 1.04 |
|  | 2 | 88.45 | 1.14 | 47.73 | 0.78 | 22.95 | 0.68 | 45.35 | 1.41 | 1.00 |
| DB12635 | 1 | 84.14 | 2.14 | 52.04 | 2.12 | 28.05 | 2.05 | 33.19 | 0.78 | 1.77 |
|  | 2 | 81.61 | 1.74 | 42.45 | 0.93 | 28.47 | 2.18 | 29.94 | 0.17 | 1.25 |
|  | 3 | 80.21 | 1.52 | 37.44 | 0.31 | 26.45 | 1.56 | 33.74 | 0.88 | 1.07 |
| DB01583 (1) | 1 | 59.86 | 1.07 | 44.72 | 0.88 | 18.45 | 1.69 | 32.16 | 1.48 | 1.28 |
| DB01583 (2) | 1 | 62.70 | 1.65 | 52.20 | 1.55 | 15.84 | 0.40 | 26.46 | 0.69 | 1.07 |
|  | 2 | 58.14 | 0.87 | 46.59 | 0.83 | 20.31 | 1.39 | 27.66 | 0.97 | 1.01 |
| DB11820 | 1 | 45.16 | 1.63 | 35.74 | 0.61 | 13.23 | 0.70 | 30.06 | 2.81 | 1.44 |
|  | 2 | 47.54 | 2.32 | 39.31 | 1.06 | 14.17 | 1.24 | 23.24 | 0.87 | 1.37 |
|  | 3 | 45.14 | 1.62 | 38.33 | 0.94 | 14.15 | 1.23 | 21.36 | 0.34 | 1.03 |
| DB00677 | 1 | 37.10 | 1.77 | 31.70 | 1.79 | 10.47 | 2.05 | 11.95 | 1.35 | 1.74 |
| Nicotinic Acid | 1 | 36.69 | 1.21 | 19.71 | 0.23 | 14.86 | 1.71 | 21.07 | 2.16 | 1.33 |
|  | 2 | 34.16 | -0.06 | 24.37 | 1.67 | 14.25 | 1.41 | 19.37 | 1.34 | 1.09 |
