# Supplementary material for: Drugs Repurposing Using QSAR, Docking and Molecular Dynamics for Possible Inhibitors of the SARS-CoV-2 Mpro Protease
Source: Molecules. 2020 Nov 6;25(21):5172. doi: 10.3390/molecules25215172 (PMC7664330; doi:10.3390/molecules25215172)
Supplement: Supplementary file 1 [file molecules-25-05172-s001.zip › Table S3.docx]

**Table S3. Estimated free energies of binding to the M^pro^ of SARS-CoV-2**

| Compound | Conformer | MM-PBSA Component | | | | | | | DELTA TOTAL |
| --- | --- | --- | --- | --- | --- | --- | --- | --- | --- |
|  |  | VDWAALS | EEL | EPB | ENPOLAR | EDISPER | DELTA G gas | DELTA G solv |  |
| DB08949 | 1 | -137.10 | -50.17 | 126.78 | -90.89 | 161.42 | -187.27 | 197.30 | 10.03 |
|  | 2 | -136.03 | -45.88 | 131.17 | -89.37 | 162.94 | -181.91 | 204.73 | 22.82 |
| DB12178 | 1 | -108.73 | -55.12 | 113.49 | -81.33 | 140.84 | -163.84 | 173.00 | 9.16 |
|  | 2 | -120.72 | -28.46 | 105.87 | -84.84 | 148.90 | -149.19 | 169.94 | 20.75 |
| DB11669 | 1 | -115.09 | -49.43 | 109.70 | -77.73 | 146.98 | -164.52 | 178.94 | 14.42 |
| DB13441 | 1 | -93.95 | -30.47 | 80.76 | -64.06 | 115.10 | -124.42 | 131.81 | 7.39 |
| DB06573 | 1 | -105.50 | -65.67 | 105.68 | -74.89 | 131.42 | -171.17 | 162.21 | -8.96 |
| DB12276 | 1 | -107.42 | -40.18 | 84.42 | -78.60 | 133.97 | -147.60 | 139.78 | -7.82 |
| DB13655 | 1 | -47.83 | -8.00 | 28.43 | -34.71 | 58.62 | -55.83 | 52.34 | -3.48 |
|  | 2 | -53.15 | -9.64 | 30.20 | -37.82 | 61.26 | -62.79 | 53.64 | -9.15 |
| DB00786 | 1 | -75.95 | -35.59 | 74.83 | -56.11 | 96.37 | -111.54 | 115.09 | 3.55 |
| DB13576 | 1 | -27.55 | 173.66 | -151.21 | -19.93 | 35.78 | 146.11 | -135.36 | 10.75 |
|  | 2 | -25.87 | 170.91 | -149.28 | -19.39 | 34.64 | 145.04 | -134.04 | 11.00 |
|  | 3 | -26.25 | 170.75 | -145.55 | -19.81 | 34.94 | 144.49 | -130.42 | 14.07 |
| DB01351 | 1 | -63.03 | -23.86 | 45.02 | -40.95 | 71.69 | -86.88 | 75.76 | -11.12 |
|  | 2 | -48.30 | -21.31 | 39.70 | -35.20 | 63.55 | -69.61 | 68.05 | -1.56 |
|  | 3 | -54.65 | -44.69 | 59.82 | -35.66 | 63.48 | -99.35 | 87.65 | -11.70 |
| DB15411 | 1 | -72.46 | -15.43 | 57.45 | -50.87 | 86.84 | -87.89 | 93.42 | 5.54 |
|  | 2 | -80.27 | -22.49 | 58.43 | -54.81 | 91.06 | -102.77 | 94.68 | -8.08 |
|  | 3 | -80.97 | -26.22 | 58.07 | -53.71 | 92.47 | -107.18 | 96.84 | -10.34 |
| DB13005 | 1 | -119.53 | -23.68 | 78.11 | -80.79 | 140.00 | -143.21 | 137.32 | -5.89 |
|  | 2 | -119.41 | -29.59 | 83.64 | -79.83 | 138.07 | -148.99 | 141.88 | -7.11 |
| DB12635 | 1 | -87.15 | 173.49 | -118.91 | -62.86 | 110.95 | 86.34 | -70.83 | 15.51 |
|  | 2 | -86.00 | 182.60 | -142.04 | -58.44 | 100.70 | 96.59 | -99.78 | -3.19 |
|  | 3 | -102.12 | 176.17 | -122.28 | -71.51 | 122.36 | 74.05 | -71.43 | 2.62 |
| DB01583 (1) | 1 | -78.32 | -64.74 | 100.38 | -51.99 | 95.43 | -143.06 | 143.82 | 0.75 |
| DB01583 (2) | 1 | -98.75 | -108.27 | 137.96 | -63.31 | 113.09 | -207.01 | 187.74 | -19.27 |
|  | 2 | -81.63 | -152.98 | 174.63 | -58.50 | 102.82 | -234.61 | 218.95 | -15.66 |
| DB11820 | 1 | -66.83 | -22.42 | 51.98 | -43.59 | 78.12 | -89.25 | 86.51 | -2.73 |
|  | 2 | -74.65 | -24.77 | 59.22 | -44.03 | 81.03 | -99.42 | 96.22 | -3.20 |
|  | 3 | -78.76 | -17.95 | 52.75 | -47.02 | 81.90 | -96.71 | 87.63 | -9.08 |
| DB00677 | 1 | -42.60 | -5.09 | 24.09 | -31.73 | 54.21 | -47.69 | 46.56 | -1.12 |
| Nicotinic Acid | 1 | -30.18 | 183.51 | -156.00 | -21.77 | 38.07 | 153.33 | -139.71 | 13.62 |
|  | 2 | -25.58 | 160.79 | -140.86 | -19.37 | 33.62 | 135.20 | -126.61 | 8.59 |
| 6Y2G | 1 | -114.32 | -166.36 | 208.40 | -76.75 | 138.32 | -280.68 | 269.98 | -10.70 |
| 6LZE | 1 | -96.81 | -83.35 | 99.85 | -64.92 | 117.42 | -180.17 | 152.35 | -27.82 |
| 6M0K | 1 | -95.14 | -89.40 | 105.05 | -64.37 | 114.41 | -184.53 | 155.09 | -29.44 |
| 7BUY | 1 | -38.16 | -9.94 | 22.72 | -30.23 | 49.57 | -48.10 | 42.05 | -6.04 |
